# Distinct Multiomic Signatures Reveal Metabolic Mechanisms of Frailty Independent of Chronological Age

**DOI:** 10.1093/geroni/igaf122.2368

**Published:** 2025-12-31

**Authors:** Noa Rappaport, Dantong Zhu, Lance Pflieger, Heeju Noh, Alex Carr, Kengo Watanabe, Max Robinson, Alice Kane

**Affiliations:** Institute for Systems Biology, Buck Institute, Phenome Health, Seattle, Washington, United States; Institute for Systems Biology, Seattle, Washington, United States; Phenome Health, Seattle, Washington, United States; Institute for Systems Biology, Seattle, Washington, United States; Institute for Systems Biology, Seattle, Washington, United States; Tokushima University Graduate School of Biomedical Sciences, Tokushima, Tokushima, Japan; Institute for Systems Biology, Seattle, Washington, United States; Institute for Systems Biology, Seattle, Washington, United States

## Abstract

Frailty is a heterogeneous clinical syndrome characterized by decline in the functioning of multiple physiological systems. Despite its prevalence, the molecular mechanisms mediating frailty remain poorly understood. This study aimed to identify biological mechanisms underlying frailty that are distinct from chronological aging by leveraging multiomic data integration in a deeply phenotyped wellness cohort. We analyzed data from >3000 participants in the Arivale program with comprehensive multiomic profiling (plasma metabolomics, proteomics, chemistries and gut microbiome) and constructed three complementary deficit accumulation frailty indices: self-reported (35 items), laboratory-based (34 items), and combined. Using a novel multiomic adapted version of the Weighted Gene Co-expression Network Analysis (WGCNA) we identified distinct multiomic modules significantly correlated with frailty independent of chronological age (p < 0.001), with notable sex-specific differences. Metabolic signatures characterized by elevated HOMA-IR, HbA1c, and triglycerides were strongly associated with frailty status, supporting the existence of an “obese frail” phenotype that challenges traditional frailty paradigms. Nutritional biomarkers, particularly low vitamin D, calcium, and potassium levels, were also significantly associated with higher frailty indices. Microbiome features explained approximately 9% of frailty variance, with butyrate production capacity inversely correlated with frailty scores. We identified specific gut microbiome enterotypes that differentially affected frailty trajectories following lifestyle intervention. Additionally, we demonstrated that lifestyle interventions significantly improved frailty trajectories, with effects moderated by polygenic risk scores for frailty. Finally, we validated our findings in external cohorts, including UKBB. Our findings reveal that frailty has distinct biological underpinnings from chronological aging, opening new avenues for personalized interventions.

